# Purtscher-like Retinopathy in a Patient with Acute Alcoholic Pancreatitis and a Literature Review

**DOI:** 10.3390/diagnostics15182317

**Published:** 2025-09-12

**Authors:** Vesela Todorova Mitkova-Hristova, Marin Anguelov Atanassov, Yumyut Remzi Idriz, Steffanie Hristova Hristova

**Affiliations:** 1 Department of Ophthalmology, Faculty of Medicine, Medical University of Plovdiv, 4002 Plovdiv, Bulgaria; marin.atanassov@mu-plovdiv.bg (M.A.A.); umitidriz952@gmail.com (Y.R.I.); 2 Faculty of Medicine, Medical University of Plovdiv, 4002 Plovdiv, Bulgaria; 21101083@mu-plovdiv.bg

**Keywords:** Purtscher-like retinopathy, Purtscher’s retinopathy, Purtscher flecken, acute pancreatitis

## Abstract

**Background and Clinical Significance**: Purtscher-like retinopathy is a rare occlusive microangiopathy that causes sudden vision loss of varying severity. It presents with diverse retinal findings, such as cotton-wool spots, haemorrhages, and optic disc and macular edema, among others. A key characteristic is the absence of trauma. This condition has been observed in patients with acute pancreatitis, renal failure, preeclampsia, HELLP syndrome, childbirth, and other systemic disorders. **Case Presentation**: A 35-year-old male presented with complaints of seeing spots in front of both eyes, with a duration of ten days following the initiation of treatment for acute alcoholic pancreatitis. On examination, best-corrected visual acuity (BCVA) in both eyes was 5/6. Fundus examination revealed multiple cotton-wool spots and haemorrhages located in the posterior pole and around the optic disc, more pronounced in the left eye, where the optic disc had blurred margins and the macular reflex was absent. Perimetry showed paracentral scotomas, and optical coherence tomography (OCT) revealed thickening and disruption of the inner retinal layers in the papillomacular region of both eyes. Fundus fluorescein angiography demonstrated adequate perfusion of the vascular network, with hypofluorescent areas in the arteriovenous phase, peripapillary and in the papillomacular zone, due to masking by cotton-wool spots and haemorrhages. Treatment included systemic antiplatelet agents, anticoagulants, and vitamins, along with topical non-steroidal anti-inflammatory drugs. Two months after the initial presentation visual acuity improved to 6/6 in both eyes. Follow-up OCT scans showed atrophy of the inner retinal layers corresponding to the previous cotton-wool spot and the areas of reduced light sensitivity on perimetry had decreased in size. **Conclusions**: Acute pancreatitis is the most common systemic condition associated with the development of Purtscher-like retinopathy. Timely diagnosis and management of the underlying systemic disease are essential for preventing ocular complications. Ophthalmological evaluation is necessary in patients with acute pancreatitis who present with visual symptoms in order to detect this often-overlooked rare condition.

## 1. Introduction

Purtscher retinopathy was first described in 1910 by Otmar Purtscher in a male patient who had sustained a traumatic brain injury following a fall from a tree. Ophthalmoscopic examination revealed characteristic fundus changes, including white ischaemic cotton-wool spots in the inner retinal layers, as well as intraretinal haemorrhages. Despite the initial loss of vision, visual acuity recovered spontaneously without any treatment [1].

In 1975, Inkeles and Walsh were the first to report a similar ophthalmoscopic presentation in three patients with acute alcoholic pancreatitis [2]. Subsequently, comparable retinal changes have been observed in other conditions, including renal failure, pancreatic adenocarcinoma, fat embolism syndrome, connective tissue diseases, bone marrow transplantation, pre-eclampsia, HELLP syndrome (Haemolysis, Elevated Liver enzymes and Low Platelets), childbirth, as well as a range of other systemic diseases. In these instances, the condition is referred to as Purtscher-like retinopathy [3]. It is believed that one of the most frequent causes of Purtscher-like retinopathy is acute pancreatitis secondary to alcohol abuse, although ocular involvement in this context is uncommon and rarely observed [3,4].

Purtscher-like retinopathy represents a rare occlusive microangiopathy, leading to unilateral or bilateral, painless, subacute visual loss of variable severity. According to a prospective observational study by Agrawal et al., conducted between April 2004 and March 2005 using the British Ophthalmological Surveillance Unit (BOSU) active reporting scheme, the incidence of the condition was found to be 0.24 per 1 million individuals [5]. However, many authors consider that the condition is underdiagnosed and likely more prevalent than reported [5,6].

The clinical presentation encompasses a range of manifestations, including cotton-wool spots, Purtscher flecken, retinal haemorrhages, and edema of the optic disc and macula. Involvement of the retina surrounding the fovea produces a picture resembling central retinal artery occlusion, with the so-called ‘pseudo-cherry red spot’. A pathognomonic sign for diagnosis is the presence of Purtscher flecken; however, these are detected in only about half of patients [7]. The characteristic retinal changes may be absent within the first 24–48 h of disease onset and typically resolve within one to three months. The prognosis is determined by the extent of retinal involvement, with macular oedema, damage to the outer retinal layers, extensive ischaemic areas, and optic disc oedema identified as adverse prognostic factors for final visual acuity [1,8]. A distinguishing feature of Purtscher-like retinopathy is the absence of preceding trauma.

In this report, we present a patient diagnosed with Purtscher-like retinopathy as a rare complication of acute alcoholic pancreatitis. Additionally, we provide a literature review of full-text articles available in Medline (National Library of Medicine/PubMed/Title/Abstract/Keywords: Purtscher-like retinopathy; Purtscher’s retinopathy; Purtscher flecken; acute pancreatitis/no search period restrictions). Articles in English were selected that provided sufficient information on the patients’ ophthalmologic status and the treatments they received, which describe cases of Purtscher-like retinopathy associated with acute pancreatitis secondary to alcohol abuse.

## 2. Case Presentation

A 35-year-old male was admitted to the University Eye Clinic at University General Hospital for Active Treatment “St. George”, Plovdiv, Bulgaria, presenting with complaints of the appearance of spots before both eyes, more pronounced in the left eye, with a duration of 10 days. The medical history indicates that over the past six months, the patient has been consuming approximately 500–600 mL of spirits with a high alcohol content (>40%) daily, as a result of experiencing a difficult divorce. The patient reported no history of systemic disease, either past or present.

For several months, he has reported morning nausea and intermittent, dull epigastric pain. Twelve days before being admitted to the Eye Clinic, he was hospitalised in the gastroenterology department of another medical facility due to the acute onset of sharp epigastric pain radiating to the back. The patient underwent a complete blood count, which revealed leukocytosis—13.11 g/L. All other hematologic parameters were within reference ranges. Fibrinogen, erythrocyte sedimentation rate, and coagulation profile were also within normal limits. Biochemical tests revealed elevated levels of: α-amylase—687 U/L; lipase—803 U/L; CRP—39.07 mg/L. Blood glucose profile was normal. Urine analysis revealed leukocyturia, proteinuria, and ketonuria. Blood pressure was measured at 110/70 mmHg with a pulse rate of 74 bp. Electrocardiogram did not show any abnormalities. Abdominal ultrasound revealed an enlarged pancreas in the region of the body with a dilated pancreatic duct. Computed tomography (CT) with oral contrast and intravenous enhancement demonstrated a small amount of free fluid within the peritoneal cavity, pelvis, and peripancreatic region; the pancreas appeared edematous, with indistinct margins and densified peripancreatic adipose tissue, and exhibited homogenous enhancement throughout all parts of the gland. Based on the patient’s medical history, clinical-laboratory and biochemical findings, corroborated by ultrasonographic and CT imaging, a diagnosis of acute alcoholic pancreatitis was established. Treatment was administered with: Ringer, No-Spa, Papaverine, Helicid, Dexofen, Fraxiparine, Meronem, Ca gluconicum, Metronidazole, Kalinor.

Due to the onset of visual complaints, the patient was evaluated by a neurologist the day after admission to the gastroenterology department. Initially, the complaints were attributed to withdrawal syndrome. No neurological deficit was detected, and the patient was referred for an ophthalmology consultation.

**Figure 1:**
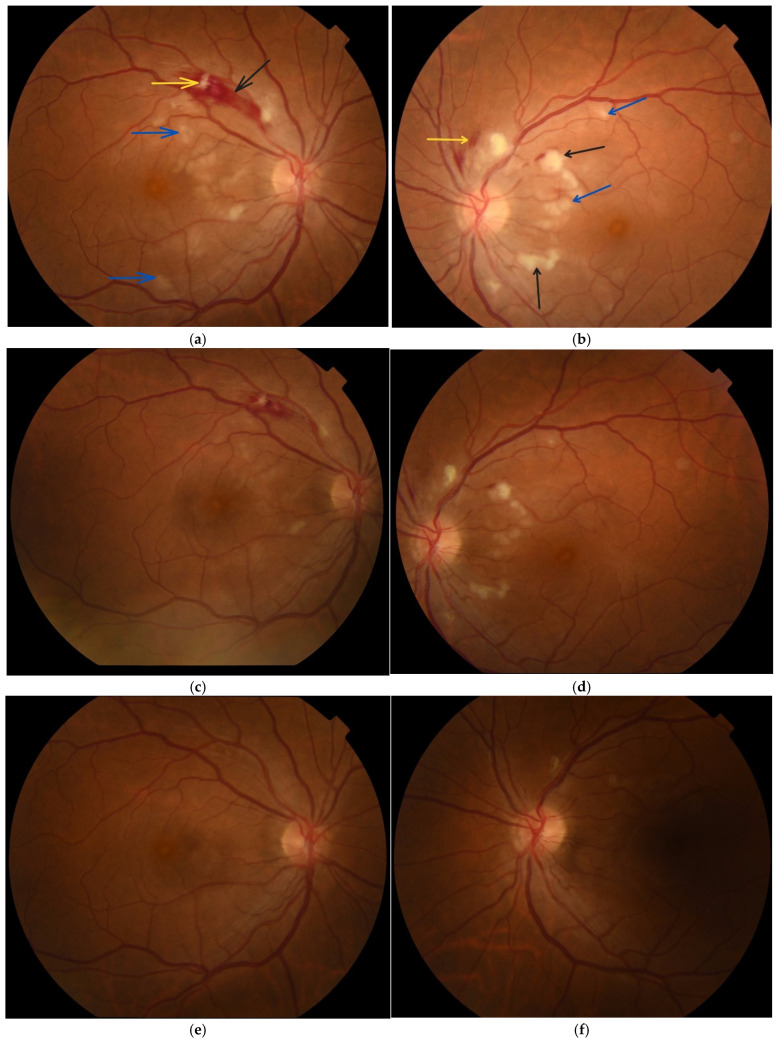
Fundus photography of both eyes: (**a**,**b**) at the time of diagnosis of Purtscher-like retinopathy, showing Purtscher fleckens (blue arrow), cotton-wool spots (black arrow), flame-shaped haemorrhages (yellow arrow), involving the posterior pole; (**c**,**d**) one week after patient follow-up—changes in the posterior pole are regressing; (**e**,**f**) two months after patient follow-up—the haemorrhages, Purtscher fleckens and cotton-wool spots are completely absorbed in the right eye and almost completely in the left. Photographic magnification at 50° 1.84×. Total observation magnification: 10× (50°). Working distance: 39 mm.

The best-corrected visual acuity (BCVA) upon admission to the ophthalmology clinic was 5/6 in both eyes. Intraocular pressure was 16 mmHg in the right eye and 14 mmHg in the left eye. Biomicroscopic examination of the anterior eye segment showed no pathological changes. Ophthalmoscopic assessment demonstrated the following findings: the optic disc of the right eye was well defined with clear margins, whereas the left optic disc exhibited mildly blurred borders. The macula of the right eye had a clear foveal reflex, which was not present in the left eye. In both eyes, flame-shaped intraretinal haemorrhages were observed along the superior-temporal vascular arcade within the nerve fibre layer, more pronounced in the right eye. In the posterior pole, particularly in the papillomacular region and peripapillary area, there were cotton-wool spots and typical Purtscher fleckens, which were more prominent in the left eye (Figure 1a,b).

**Figure 2:**
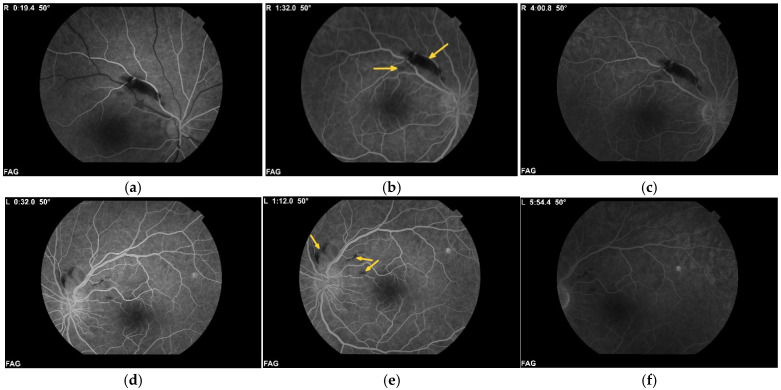
Fundus fluorescein angiography at the time of diagnosis of Purtscher-like retinopathy, demonstrating satisfactory perfusion of the vascular network in both eyes ((**a**–**c**)—right eye; (**d**–**f**)—left eye), with areas of non-perfusion attributed to masking by haemorrhages and intraretinal exudates (yellow arrows). Photographic magnification at 50° 1.84×. Total observation magnification: 10× (50°). Working distance: 39 mm.

Fundus fluorescein angiography (FFA) demonstrated satisfactory perfusion of the retinal vascular network in both eyes, with focal areas of absent perfusion corresponding to regions of haemorrhages and wet exudates—an effect of masking (Figure 2).

**Figure 3:**
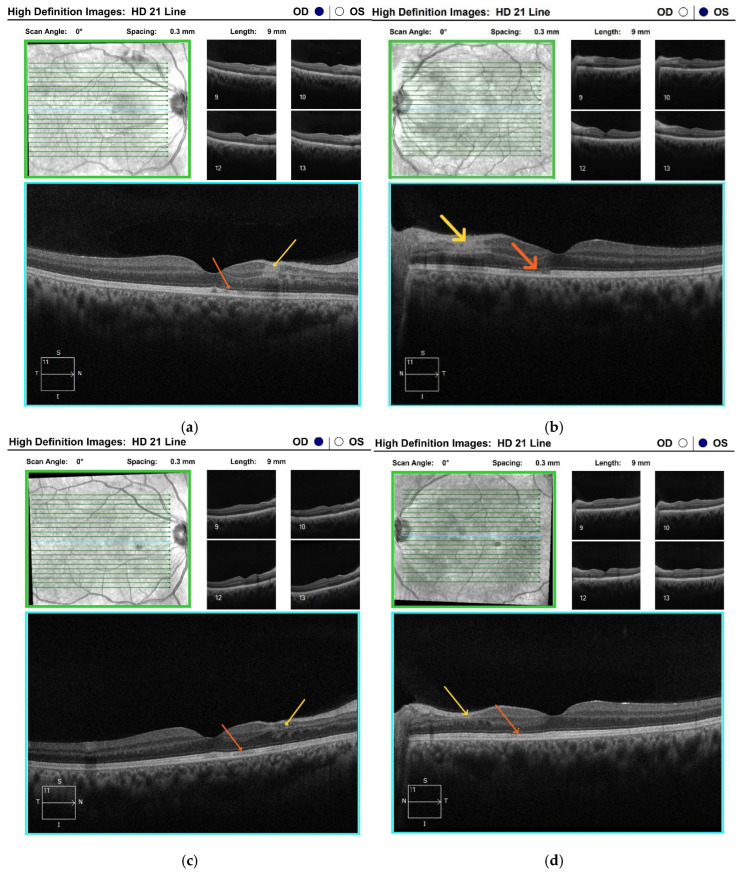

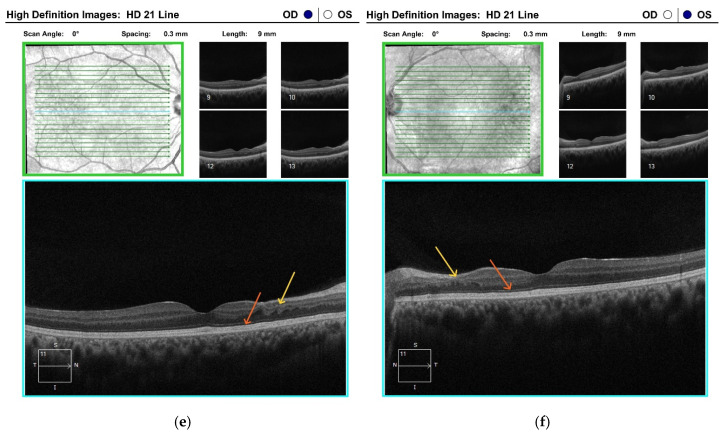
Optical coherence tomography of the maculae of both eyes: (**a**,**b**) at the time of diagnosis of Purtscher-like retinopathy—reveals thickening, hyperreflectivity, and loss of definition of the inner retinal layers, corresponding to ischaemic areas and Purtscher flecken (yellow arrow); in the outer retinal layers, a paramacular, nasal disruption of the photoreceptor layer (orange arrow) is observed, more pronounced in the left eye (**b**); (**c**,**d**) two months after follow-up—the paramacular, hyperreflective areas in the inner retinal layers demonstrate atrophy and thinning in the affected zones (yellow arrow); in the outer layers, partial restoration of the ellipsoid zone is seen, with persistent disruption of the line corresponding to the junction between the inner and outer segments of the photoreceptors (orange arrow); (**e**,**f**) nearly two years after follow-up—no progression of findings in either eye (yellow and orange arrows). HD 21 Line scan, line length 9 mm, scan angle 0°, inter-line spacing 0.3 mm. Scan volume: 9 × 6 × 2 mm. Horizontal scale ≈ 8.8 µm/pixel; vertical scale ≈ 1.95 µm/pixel; inter-line scale = 0.3 mm.

Optical coherence tomography (OCT) of the maculae revealed alterations in both the inner and outer retinal layers, with more pronounced changes in the left eye (Figure 3a,b).

**Figure 4:**
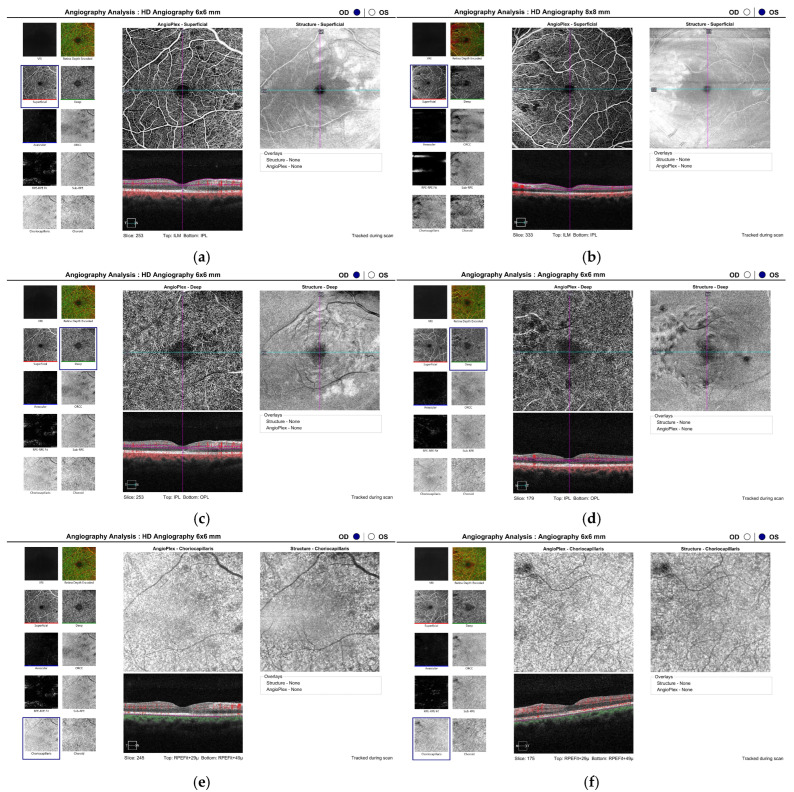
Angio-OCT of both eyes at the time of diagnosing Purtscher-like retinopathy: (**a**,**b**) illustrate the superficial plexus, indicating preserved perfusion; (**c**,**d**) illustrate the deep plexus, revealing areas of hypoperfusion in the papillomacular region of both eyes and an enlarged, slightly deformed nasal foveolar avascular zone in the left eye (**d**); (**e**,**f**) illustrate the choroidal capillaries, which have preserved perfusion. HD OCT angiography of the macula (ZEISS Cirrus 6000, 6 × 6 mm). The magnification and calibrated scale allow precise measurements of the capillary network and FAZ.

Analysis with angio-OCT (OCT-A) demonstrated preserved perfusion in the superficial plexus (Figure 4a,b), whereas zones of hypoperfusion were visualised in the deep plexus within the papillomacular region of both eyes (Figure 4c,d). The perfusion of the choriocapillaris was normal in the early stages of the disease (Figure 4e,f).

**Figure 5:**
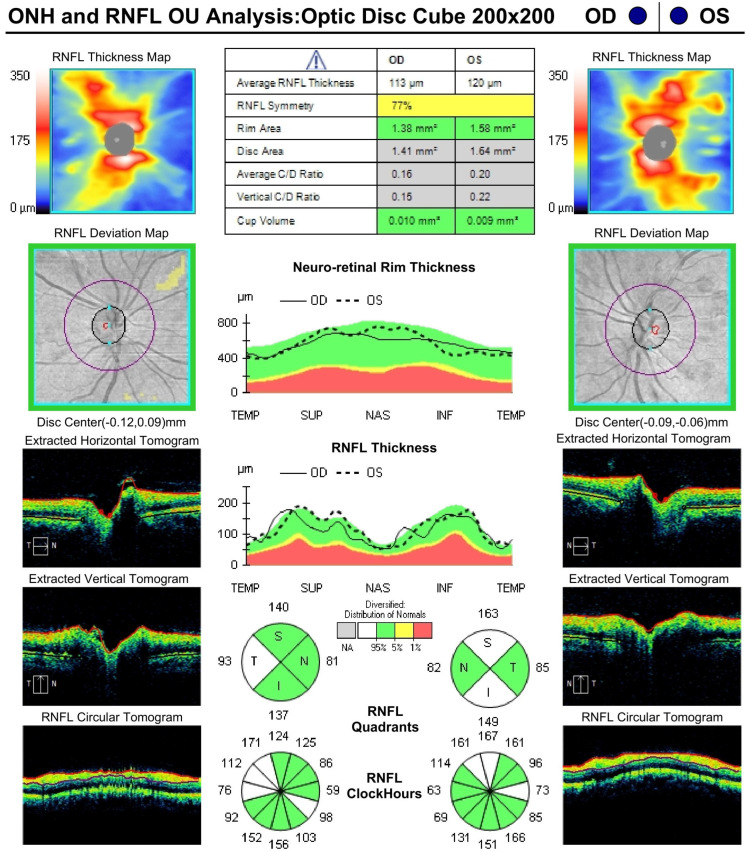
Optical coherence tomography of the optic nerves at the time of diagnosis of Purtscher-like retinopathy—reveals mild thickening of the nerve fibre layer, more pronounced in the left eye.

OCT analysis of the optic nerves revealed mild thickening of the nerve fibre layer, more pronounced in the left eye (Figure 5).

**Figure 6:**
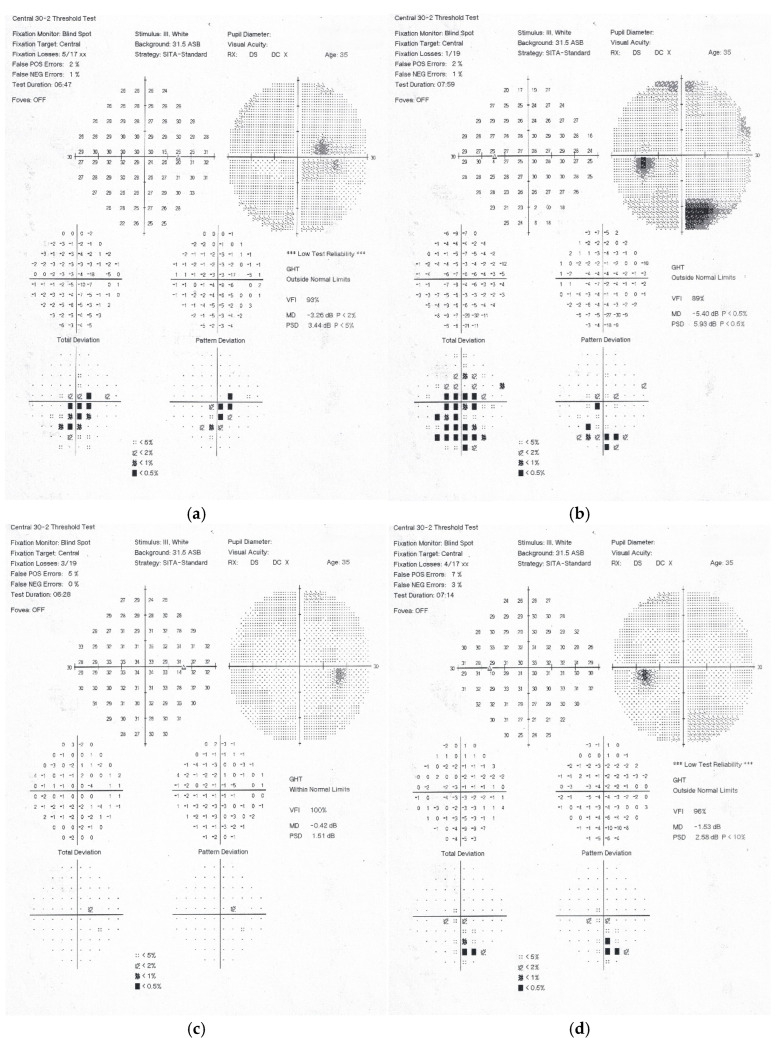
Computerised perimetry (Central 30-2 Threshold Test, HFA) of both eyes at the time of diagnosis of Purtscher-like retinopathy: (**a**,**b**) illustrate areas of decreased light sensitivity centrally and paracentrally in the inferior half of the visual fields of both eyes; (**c**,**d**), two months after follow-up, demonstrate a reduction in the regions of decreased light sensitivity.

Computerised perimetry identified areas of reduced light sensitivity centrally and paracentrally in the inferior hemifield, corresponding to more pronounced ophthalmoscopic changes in the superior half of the retinae (Figure 6a,b).

During the follow-up, clinical, laboratory, and biochemical tests demonstrated a significant decrease in the levels of α-amylase (203 U/L) and lipase (225 U/L), although these levels remained above the reference range. In terms of differential diagnosis, HIV-associated retinitis was excluded by a HIV-ELISA Rapid test, which yielded a negative result. Computed tomography of the head demonstrated no pathological changes. In the differential diagnosis we also considered hypertensive retinopathy; however, the patient had no evidence of cardiovascular abnormalities. Normal blood glucose levels excluded diabetic retinopathy as a potential cause of the fundoscopic findings. Another condition that can present with a similar clinical picture is systemic lupus erythematosus. The absence of systemic manifestations (skin and mucosal changes, musculoskeletal involvement, renal or pulmonary disease, anemia, etc.) led us to exclude it as a likely etiology.

Based on the history of acute alcoholic pancreatitis and the presence of characteristic ophthalmoscopic findings, we diagnosed Purtscher-like retinopathy. We administered treatment with vasodilator medications—Pentilline and Milgamma N, both systemically and locally, using a non-steroidal anti-inflammatory drug (NSAID) and Neuretin.

Two months following the initial examination, the patient’s visual acuity was 6/6 in both eyes. Haemorrhages, cotton-wool spots and Purtscher fleckens had fully resolved in the right eye and were almost completely resorbed in the left (Figure 1e,f). On OCT, the paramacular hyperreflective areas in the inner retinal layers had progressed to atrophy and thinning in the affected regions. Partial restoration of the ellipsoid zone was observed in the outer retinal layers (Figure 3c,d). Follow-up computerised perimetry demonstrated a reduction in areas of decreased light sensitivity (Figure 6c,d).

Over an almost two-year follow-up period, the patient’s visual acuity remained at 6/6, with no progression observed in the OCT findings (Figure 3e,f).

## 3. Discussion

The precise mechanism of Purtscher-like retinopathy is still not fully understood, but the existing evidence indicates that its origin is embolic [1]. Elevated lipase levels observed in acute pancreatitis are considered a key pathogenic factor. They induce fat embolization of precapillary arterioles, which is subsequently followed by complement activation and the formation of the C5a component, which is a potent mediator of inflammation and immune response. The release of activated proteases, such as trypsin, also results in complement activation. This, in turn, induces granulocyte aggregation and the formation of micro-occlusions. The resulting ischaemia within the microcirculatory bed underlies the characteristic ophthalmoscopic findings [6,9]. The most common retinal alterations include cotton-wool exudates and intraretinal haemorrhages; notably, the pathognomonic Purtscher fleckens may be absent in some patients [5]. Purtscher fleckens are characterised by multiple, well-demarcated, polygonal white spots situated within the superficial layers of the inner retina, located between arterioles and venules. Their size ranges from one quarter to several optic disc diameters, with each spot encircled by an area of normal retina [1,5]. In our case, the spots were smaller in size, suggesting occlusion of the distal segments of the arterioles. The alterations were predominantly localised to the posterior pole and the papillomacular region, and resolved within two months. The restriction of lesions to the posterior pole is likely associated with the particularities of the blood supply in the peripapillary and macular areas. In addition to the dual-layered retinal capillary network, a superficial capillary plexus is present in the peripapillary nerve fibre layer surrounding the optic nerve. In this region, the capillaries are longer, run more linearly, and form fewer anastomoses, rendering them more susceptible to embolic occlusion and ischaemia. Furthermore, the fluid nature of lipids likely aids the penetration of fat emboli into capillaries with smaller diameters [1,9]. Purtscher fleckens should be distinguished from cotton-wool spots, which are characterised by indistinct borders and a superficial location over the major retinal vessels [10,11]. In all the case reports we reviewed involving patients with acute alcoholic pancreatitis, the presence of typical Purtscher fleckens was documented (Table 1) [3,4,8,9,10,11,12,13,14,15,16,17,18,19,20].

Male sex and pancreatitis as etiological factors in Purtscher-like retinopathy are associated with a better visual outcome [14]. It is likely that, due to their smaller size, fat emboli in some cases do not cause vascular occlusion. In the study by Serhan et al., which encompassed 168 cases of Purtscher and Purtscher-like retinopathy, the incidence of the condition among patients with acute pancreatitis was found to be similar in both sexes. The authors observed that the condition most frequently presents in individuals in their fifth and sixth decades of life [7]. Our literature review demonstrates a higher incidence among males—87.5%—as well as a younger mean age at onset (32.33 ± 8.2 years). This is likely attributable to the fact that our analysis included only patients with acute pancreatitis induced by alcohol abuse; these findings may be explained by the higher prevalence of alcohol consumption within this age group (see Table 1).

As reported in the study by Serhan et al., the most common symptom observed in Purtscher-like retinopathy is blurred central vision, with complaints more frequently affecting the left eye [7]. In our patient, the symptoms were also more pronounced in the left eye, where a more severe clinical finding was observed. This may be explained by the forced position adopted during acute pancreatitis—patients remain lying on their left side for prolonged periods to alleviate systemic complaints. Such positioning could hypothetically impair venous drainage from the left eye, particularly in the presence of hypovolaemia, vascular compression, or increased intra-abdominal pressure.

In our case, pathological changes were present in both eyes. According to the study by Agrawal and McKibbin, bilateral involvement was observed in all patients with acute pancreatitis, in contrast to cases with traumatic aetiology, where bilaterality was found in 60% of cases [5]. Based on our review of the literature, among all reported cases of acute alcoholic pancreatitis [3,4,8,9,10,11,12,13,14,15,17,19,20], only one presented with unilateral Purtscher-like retinopathy [16] (see Table 1).

Visual impairments in Purtscher and Purtscher-like retinopathy typically manifest within 24 to 48 h following the onset of the associated condition and can range from minimal to significant visual impairment [1]. In our case, as well as in reports by other authors [3,10,12,15,18,20,21], visual disturbances emerged by the second day after the onset of acute alcoholic pancreatitis. In the initial hours post-hospitalisation, visual complaints are frequently misattributed to alcohol intoxication or withdrawal, which may result in delayed or missed diagnosis [15].

According to the literature reviewed, more than half of patients present with binocular visual acuity reduced to ‘counting fingers’ [4,8,10,12,15,17,21]; in one case, this was observed in only one eye [11], while another patient exhibited visual acuity of ‘hand movements’ in both eyes [9]. In our patient, visual acuity was only minimally reduced, which is likely attributable to the rapid establishment of the diagnosis and the prompt initiation of treatment.

Atrophy of the retinal pigment epithelium, as well as alterations in the outer retinal layers, provide evidence of choroidal vascular network involvement during the acute phase of the disease. Supporting this, indocyanine green angiography demonstrates hypofluorescent areas in the affected choroidal regions [1,5]. Hay et al. investigated the functional status of the retina using electroretinography in a patient with Purtscher-like retinopathy secondary to acute alcoholic pancreatitis, and observed reduced amplitudes of both the A-wave and B-wave, even after the resolution of retinal edema. These results reflect a lasting functional loss affecting both the inner and outer layers of the retina [21]. The choriocapillaris in our patient did not demonstrate areas of ischemia on OCT-A, which likely explains the subtle changes observed in the outer retinal layers as well as the preserved visual acuity.

Optical coherence tomography in our patient at the onset of the disease revealed thickening, hyperreflectivity, and loss of definition in the inner retinal layers—indicative of ischaemia of the inner retinal circulation. Nevertheless, OCT-A demonstrated preserved perfusion in the superficial plexus, whereas small areas of capillary hypoperfusion and a slight nasal enlargement of the foveal avascular zone in the left eye were observed in the deep plexus. Similar OCT findings have been reported by other authors in patients with Purtscher-like retinopathy, emphasising that OCT-A can effectively replace invasive FFA for the detection of retinal ischaemic alterations, even at an early stage [22]. In our case, microischaemic alterations in the deep plexus were not visualised on FFA.

Macular atrophy and disruption of the IS/OS junction, as observed on OCT, are considered unfavourable prognostic indicators [6,22]. Although IS/OS junction alterations were also observed in our patient, central visual acuity in both eyes was fully restored, as the lesions were located outside the fovea. Alaei et al. describe an intriguing case of pancreatitis-induced Purtscher-like retinopathy in a pregnant woman following excessive use of gastric acid-suppressing medications. During follow-up, they identified an OCT finding similar to ours—reduced retinal thickness in the outer layers due to photoreceptor injury [23]. According to the authors, the final functional outcome of the condition ranges from complete recovery to permanent visual loss, underscoring the importance of early detection and prompt initiation of therapy. Nevertheless, there is currently a lack of compelling evidence supporting the efficacy of any of the treatments employed [6].

The unclear pathophysiology and rarity of the cases contribute to the absence of an established therapeutic strategy [14]. In most instances, acute lesions resolve spontaneously within 1–3 months following their onset. The literature describes successful outcomes with intravenous administration of high-dose corticosteroids. Their mechanism of action involves stabilisation of cellular membranes and compromised microcirculatory channels, as well as facilitating partial recovery of neuronal fibres that have not undergone irreversible damage [11]. Furthermore, corticosteroids inhibit complement activation and the formation of leukocyte aggregates, suppress the production of free oxygen radicals, and improve microcirculation in certain patients [6,20]. In the presence of macular edema, they exert a pronounced antiedematous effect [24]. Wieczorek et al. reported significant improvement in macular edema and visual acuity in a patient with acute alcoholic pancreatitis and Purtscher-like retinopathy following intravenous administration of hydrocortisone [3]. In our case, systemic corticosteroid therapy was not administered, as the patient’s baseline visual acuity was high and potential systemic and ocular adverse effects were considered. The likely uncertain outcomes associated with systemic corticosteroid treatment may explain why such therapy was reported in only three patients among the cases we reviewed in the literature [3,4,11]. Gahn et al. report the use of parabulbar and intravitreal corticosteroids in a patient with bilateral, very low visual acuity—counting fingers; however, the improvement achieved was minimal—20/400 [17].

One of the mechanisms of action of non-steroidal anti-inflammatory drugs (NSAIDs) is the inhibition of cyclooxygenase enzymes, which are involved in the synthesis of prostaglandins—mediators of the inflammatory process and vasodilation. Consequently, they are also regarded as a suitable therapeutic option for patients with Purtscher-like retinopathy. In our case, the patient had already received systemic therapy with NSAIDs during the initial hospitalisation in the gastroenterology department, which is why we administered only a topical NSAID preparation.

The application of vasodilators and antiplatelet agents in this disease also lacks definitively proven efficacy. Nevertheless, we included Pentillin in our therapeutic plan because it improves microcirculation through its combined vasodilatory and antiplatelet properties. Its use in Purtscher-like retinopathy has also been reported by other authors [3,25].

## 4. Conclusions

Acute pancreatitis is the most common systemic disease associated with the development of Purtscher-like retinopathy, with alcohol abuse being the leading aetiological factor. The condition is more commonly observed in men and usually affects both retinas.

The clinical case presented underscores the necessity for heightened vigilance regarding visual complaints in patients with acute pancreatitis resulting from alcohol abuse. In the presence of such symptoms, ophthalmological evaluation should be conducted at an early stage of the disease to facilitate timely recognition of this rare but frequently underestimated condition. Early diagnosis, meticulous follow-up, and appropriate management of the underlying disease are essential to minimise the risk of permanent visual impairment and to achieve better functional outcomes.

## Figures and Tables

**Table 1:** Alcohol pancreatitis-related Purtscher-like retinopathy cases in the literature.

Author Year of Publication	Age	Sex	Laterality	Initial VA	Final VA	Ocular Finding	Onset of OC (Day)	Treatment
Wieczorek M [3] 2021	32	M	BL	5/40	8/40	PF, CWS, ME	2	Pentoxifylline NSAIDs Systemic steroids
Tariq T [20] 2019	37	F	BL	RE—20/100 LE—20/200	20/20	PF, CWS, ME	1	For pancreatitis
Sharma S [8] 2023	28	M	BL	CF	RE—6/18 LE—6/60	PF, CWS, RH, ME	3	For pancreatitis Oral antibiotic
Buckley A [9] 1996	52	M	BL	HM	RE—6/9 LE—6/60	PF, CWS, RH	-	-
Campo S [13] 2000	32	M	BL	RE—1/10 LE—2/50	RE—8/10 LE—7/10	PF, CWS, RH	-	For pancreatitis
Haq F [21] 2002	20	M	BL	CF	RE—CF LE—20/40	PF, CWS, RH	1	-
Almpani M [15] 2023	33	F	BL	CF	RE—20/60 LE—20/100	PF, RH	2	For pancreatitis
Subudhi P [4] 2016	28	M	BL	CF	RE—20/80 LE—20/120	PF, CWS, RH	-	Systemic steroids
Nema N [10] 2016	23	M	BL	CF	RE—6/18 LE—6/9	PF, CWS, RH	2	For pancreatitis
Gahn G [17] 2020	22	M	BL	CF	20/400	PF, CWS, RH, ME	3	Intravitreal, parabulbar steroids
Prabhu V [11] 2024	33	M	BL	RE—6/120 LE—CF	RE—6/18 LE—6/300	PF, CWS, RH	21	Systemic steroids
Carrera C [12] 2005	35	M	BL	CF	RE-20/20 LE—20/100	PF, CWS, RH	1	For pancreatitis
Narang S [18] 2022	30	M	BL	RE—20/200 LE—20/100	20/30	PF, CWS, RH	1	For pancreatitis
Dholakia S [16] 2013	43	M	UL	-	-	PF, RH	4	For pancreatitis
Ciortescu I [19] 2011	37	M	BL	RE—1/20 LE—1/50	-	PF, CWS, RH	-	-
Mitkova-Hristova V [the present study] 2025	35	M	BL	5/6	6/6	PF, CWS, RH	2	Pentoxifylline NSAIDs Oral antibiotic

BL—bilateral; CF—count finger; CWS—cotton-wool spot; F—female; HM—hand movements; LE—left eye; M—male; ME—macular edema; NSAIDs—non-steroidal anti-inflammatory drugs; OC—ocular complaint; PF—Purtscher flecken; RE—right eye; RH—retinal haemorrhage; UL—unilateral; VA—visual acuity.

## Data Availability

The data presented in this study are available on request from the corresponding author due to privacy.
